# Highly Efficient Biosynthesis of Glycyrrhetinic Acid Glucosides by Coupling of Microbial Glycosyltransferase to Plant Sucrose Synthase

**DOI:** 10.3389/fbioe.2021.645079

**Published:** 2021-06-08

**Authors:** Mohamed Yassin Ali, Qing Chang, Quande Yan, Zheng Qian, Xiang Guo, Kieran Thow, Jinhong Wu, Yong Zhang, Yan Feng

**Affiliations:** ^1^State Key Laboratory of Microbial Metabolism, Joint International Research Laboratory of Metabolic and Developmental Sciences, School of Life Sciences and Biotechnology, Shanghai Jiao Tong University, Shanghai, China; ^2^Biochemistry Department, Faculty of Agriculture, Fayoum University, Fayoum, Egypt; ^3^Department of Food Science and Technology, School of Agriculture and Biology, Shanghai Jiao Tong University, Shanghai, China

**Keywords:** glycosylation, glycyrrhetinic acid, UDP-glycosyltransferases, biocatalytic cascade, sucrose synthase

## Abstract

Glycyrrhetinic acid (GA) is a principal bioactive pentacyclic triterpenoid from *Glycyrrhiza uralensis*. Uridine diphosphate-dependent glycosyltransferases (UGTs) have been widely used to catalyze glycosylation of diverse nature products for the development of potential therapeutic compounds. In this study, we have characterized a UGT109A3 from *Bacillus subtilis*, which can glycosylate both the free C3 hydroxyl and C30 carboxyl groups of GA to yield a unique 3, 30-O-β-D-diglucoside-GA. By coupling the microbial UGT109A3 to plant sucrose synthase (SUS), GA-diglucoside could be biosynthesized in an efficient and economical way. With a fed-batch glycosylation, a large scale of GA-diglucoside (6.26 mM, 4.98 g/L in 8 h) could be enzymatically transformed from GA. The obtained GA-diglucoside showed a significant water solubility improvement of around 3.4 × 10^3^ fold compared with that of the parent GA (29 μM). Moreover, it also exhibited dose-dependent cytotoxicity toward human colon carcinoma Caco-2 cell line according to MTT assay, having an IC_50_ at 160 μM. This study not only establishes efficient platform for producing GA-glucosides, but is also valuable for developing further the biosynthesis of other complex glycosylated natural products.

## Introduction

In nature, glycosylation has been demonstrated to be a powerful strategy for providing the structure and function diversity of natural products to fulfill a vast range of physiological roles in cellular function and survival (Lairson et al., 2008). Glycosylation is a dynamic and complicated molecular modification that involves the action of multiple enzymes. Among the glycosylation enzymes, glycosyltransferases catalyze the transfer of sugar moieties from the activated sugar donors to specific acceptors and are responsible for the majority of glycosidic bond formation in cells (Breton et al., 2006; Rini, 2009). Uridine diphosphate (UDP)-glycosyltransferases (UGTs) belongs to the largest glycosyltransferase family 1 and are capable of catalyzing glycosylation of various lipophilic molecules with the nucleotide UDP-sugars as the donor (Schmid et al., 2016). Several UGTs have been exploited to alter the glycosylation patterns and sugar structures of natural products for developing potential therapeutic compounds (Lairson et al., 2008; Xu and Kong, 2020; Ali et al., 2021). The conjugated glycosyl groups usually could alter water solubility, stability, and pharmacological properties of natural products (Kren and Martínková, 2001).

Glycyrrhetinic acid (GA) is a typical pentacyclic triterpenoid from roots and rhizomes extracts of herb licorice species possessing significant bioactivities such as anti-inflammatory, anti-tumor, and hepatoprotective activities (Wu et al., 2015; Zhao et al., 2017). However, the chemical structure of GA consists of a large non-polar pentacyclic triterpenoid skeleton, which leads to poor water solubility, potentially weakening its bioavailability and practical applications (Gauthier et al., 2011; Alqahtani et al., 2013; Guo, 2017). Glycosylation is a promising approach for improving the bioavailability and pharmacological potency of GA. Several UGTs have been characterized and their ability to produce valuable GA glycoside derivatives investigated, including microbial BsYjiC from *Bacillus subtilis* 168 (Dai et al., 2018a), plant UGT73C11 from *Barbarea vulgaris* (Liu et al., 2017), and GuUGT from *Glycyrrhiza uralensis* (Huang et al., 2019). Due to their rapid and simple recombinant protein productions, microbial UGTs generally are regarded as more promising biocatalysts for scale-up glycosylations of structurally diverse natural products (Chen et al., 2018; Dai et al., 2018b; Huang et al., 2019).

In this study, we have characterized a UGT from *B. subtilis*, designated as UGT109A3, which showed catalytic activity toward GA. By using high-performance liquid chromatography–electrospray ionization–mass spectrometry (HPLC–ESI–MS) and nuclear magnetic resonance (NMR), we noted that UGT109A3 could transfer the glucosyl moieties to the free C-3 OH and C-30 COOH groups of GA to yield a specific GA-diglucoside, 3, 30-O-β-D-diglucoside-GA. As sucrose synthase (SUS) is capable of catalyzing a regeneration of the costly sugar donor UDP-glucose (UDPG) (Terasaka et al., 2012), an artificial biocatalytic cascade by coupling microbial UGT109A3 to plant SUS was developed, optimized, and applied to synthesize GA-diglucoside efficiently. The GA-diglucoside exhibited an almost 3.4 × 10^3^-fold water solubility improvement compared with that of GA.

## Materials and Methods

### Strains, Plasmid, and Chemicals

Uridine diphosphate, UDPG, GA (>98.0% purity), and dimethyl sulfoxide (DMSO) were purchased from Sigma-Aldrich (St. Louis, MO, United States). Sucrose was purchased from Raygood Biotech (Shanghai, China). *Escherichia coli* DH5α and BL21 (*DE3*) chemically competent cells were purchased from Xingeron (Shanghai, China). All other reagents were the high purity available.

### Sequence Alignment and Phylogenetic Analysis

The amino acid sequences of the selected UGTs catalyzing glycosylation of secondary metabolites were aligned using ClustalW (Thompson et al., 1994). A maximum likelihood non-rooted phylogenetic tree was constructed by MEGA 7.0.14^1^. Bootstrap value was set at 1000 replications.

### Heterologous Expression and Purification of Glycosyltransferases

*Escherichia coli* codon-optimized genes encoding *Bacillus* UGT109A3 and *Arabidopsis thaliana* SUS (GenBank accession no. NM_122090.4) were synthesized and cloned into pET28a expression vectors (Supplementary Figure 1). The fidelity of inserting fragments in pET28a was confirmed by sequencing. The constructed expression vectors of pET-UGT109A3 and pET-SUS were chemically transformed into *E. coli* BL21 (*DE3*) competent cells, respectively. The transformed cells were cultivated in 50 mL Luria-Bertani (LB) medium supplemented with 50 μg/mL kanamycin with shaking at 220 rpm, 37°C overnight. The initial culture was inoculated into 1 L auto-inducing liquid medium (Studier, 2005) and cultured at 37°C and 220 rpm until the OD_600_ reached approximately 2.0. After culture for additional 18 h at 20°C, cells were harvested by centrifugation. The harvested cells were resuspended in 20 mM Tris–HCl pH 8.0, 500 mM NaCl, and 20 mM imidazole buffer and lysed by homogenizer at 4°C. The lysed cells were centrifuged at 12,000 rpm for 30 min, and the supernatant was loaded on a Ni-NTA agarose column (Smart-lifesciences, Changzhou, China). After processing with a washing buffer of 20 mM Tris–HCl pH 8.0, 500 mM NaCl, and 50 mM imidazole, recombinant proteins were eluted with a buffer of 20 mM Tris–HCl pH 8.0, 500 mM NaCl, and 200 mM imidazole and concentrated by centrifugation at 4°C using Amicon Ultra-15 tubes (Millipore, United States). A 12% SDS-PAGE gel electrophoresis was used to examine the purified proteins. Concentrations of proteins were determined using BCA protein assay (Pierce, United States) with bovine serum albumin as the standard.

### Enzymatic Activity Determination

The glycosylation was analyzed using the high-performance liquid chromatography (HPLC) method (Tan et al., 2019). The standard glycosylation reaction was performed in 250 μL reaction buffer, containing 20 mM Tris–HCl pH 8.0, 1 mM GA dissolved in DMSO, 5 mM UDPG, and 10 μg UGT109A3. After incubation at 35°C for 12 h, the reaction was quenched by adding an equal volume of n-butanol and then analyzed by HPLC or HPLC-ESI-MS. The sample was injected on an Agilent1260 HPLC, with UV detection at 254 nm, using an Agilent Eclipse XDB-C18 column (4.6 × 150 mM, 5 μm particles) at 40°C with a flow rate of 0.8 mL/min. Linear gradient elution was performed with water containing 0.1% formic acid (A) and acetonitrile (B) as the mobile phases. The following program was applied: 0–3.5 min, 79% B; 3.5–8.2 min, 79–98% B; 8.2–11 min, 98–79% B; and 11–25 min, 79% B.

The SUS activity was determined in 250 μL reaction buffer containing the mixture contained 50 mM Tris–HCl pH 7.5, 10 mM UDP, and 500 mM sucrose, 10 μg protein (Ali et al., 2021). After incubation for 1 h at 35°C, the reactions were terminated by heating at 95°C for 10 min. The supernatant was archived by centrifugation at 10,000 × *g* for 20 min and applied to the HPLC system. The Eclipse XDB-C18 column (4.6 × 250 mM, Agilent) was equilibrated with 100 mM Na_2_HPO_4_/NaH_2_PO_4_ pH 6.5, and 10 mM tetrabutylammonium bromide. The components were separated at a velocity of 1 mL/min. The yield of UDPG was monitored at 254 nm and was assigned based on the retention time of a standard.

### Enzyme Kinetic Parameter Measurement

The kinetic parameters of UGT109A3 were determined by varying the concentration of UDPG or GA in the reaction mixtures, 50 mM Tris–HCl, pH 8.0, 1 μL 1% (v/v) Tween-80 and 10 μM UGT109A3 enzyme, final volume of 300 μL. To investigate the kinetic parameters of UGT109A3 toward UDPG, the concentration of UDPG was varied from 0.2 to 2 mM, with the GA concentration fixed at 1 mM; and kinetic parameter of UGT109A3 toward GA was also analyzed by varying the GA concentration from 0.1 to 2.5 mM, with the UDPG concentration fixed at 2 mM. The reactions were incubated at 35°C for 15 min. The reactions were terminated by adding the same volume of n-butanol. HPLC analysis was performed to quantify the conversion. The GA-glucoside amounts were monitored at 254 nm and were assigned based on the retention time. The kinetic parameters were obtained by fitting initial velocity data to the Michaelis–Menten equation using GraphPad Prism 5.0 (GraphPad Inc.).

### Structural Analysis of GA Derivatives

To characterize the structures of GA derivatives, the glycosylated products were purified by a preparative HPLC system coupled with a preparative reverse-phase C18 column (21.2 × 250 mm, 5 μm particle, Welch, Shanghai, China). The preparative column was eluted with distilled water (solvent A) and methanol (solvent B) using a gradient program of 40–100% B in 0–60 min. The purified fractions containing target GA glucosides were pooled and concentrated using the rotary evaporator and vacuum freeze drier. The dried powders were dissolved in methanol-d4 (Sigma-Aldrich). The compounds were further characterized with a 900-MHz Avance II 900 (Bruker, Germany) BioSpin NMR spectrometer.

### Construction and Optimization of UGT109A3-SUS Biocatalytic Cascade

A biocatalytic cascade of UGT109A3-SUS was constructed by using a multiple component mixture of 50 μg/mL UGT109A3 and 20 μg/mL SUS, 1 mM GA, 0.5 mM UDP, 20 mM Tris–HCl pH 7.5, and 500 mM sucrose. The optimal ratio of UGT109A3 to SUS was determined by varying the ratio of UGT109A3 to SUS to examine conversion rates. The effects of varying Tris–HCl buffer pH and temperature on the cascade reaction were examined, respectively. The effect of sucrose, UDP, and DMSO effects on the UGT109-SUS biocatalytic cascade reaction were investigated by determination of GA glycosylation.

### Fed-Batch Conversion

Reaction mixtures (100 mL) containing 1 mM GA, 0.5 mM UDP, 50 mM Tris–HCl pH 7.5, 500 M sucrose, 50 μg/mL UGT109A3, and 20 μg/mL SUS were incubated at 35°C. The reaction tubes were gently shaking at 150 rpm. An aliquot of 200 μL reactant was collected and quenched by adding an equal volume of n-butanol. After centrifugation at 12,000 × *g* for 15 min, the supernatant was filtered through 0.22 μm filters and subjected to HPLC analysis. The 200 mM GA was periodically supplemented to the reaction mixtures. Fresh enzyme (50 μg/mL UGT109A3, and 20 μg/mL SUS) was added after 4 h from reaction start.

### Water Solubility and Cytotoxic Determination

The water solubility of compounds were determined as previously described (Liu et al., 2017). Excess amounts of GA or GA-diglucoside were added to distilled water. The suspensions were extensively mixed by vortexing for 2 h at 25°C and centrifuged at 15,000 × *g* for 30 min to remove insoluble materials. The supernatant solutions were collected and pretreated with n-butanol as previously described, before subjecting to HPLC analysis. The HPLC peaks at 254 nm were integrated to calculate the sample solution concentrations.

Cytotoxic activities of compounds against several cell lines were evaluated using the MTT assay (Jiao et al., 1992; Liu et al., 1997). A 96-well plate was seeded with 10^3^ cells in 100 μL DMEM medium supplemented with 10% FBS and allowed to attach for 24 h. Cells were treated with GA or GA-glucoside at concentration of 5, 30, and 100 μM, and incubated for 48 h. Each treatment was conducted in duplicate. After the treatment, compounds containing media were removed and washed with 200 μL PBS. In each well, 20 μL methyl-thiazol-tetrazolium (MTT, 5 mg/mL) reagent was added and incubated for 4 h at 37°C. The MTT reagent was removed, and 150 μL DMSO was added to each well. The optical density was measured by a microplate reader at 490 nm. SPSS 16.0 software was used to calculate the IC_50_ values of the compounds (Zhang et al., 2016).

## Results and Discussion

### Sequence Alignment and Phylogenetic Analysis

DNA sequencing projects have provided hundreds of thousands of protein-coding sequences from prokaryotic and eukaryotic organisms. Microorganisms have a wealth of UGTs involved in glycosylating diverse metabolites or exogenous chemicals (Yang et al., 2018). By checking available microbial genomic sequences, a putative glycosyltransferase from *B. subtilis* (GenBank: NLS39232.1) comprising 392 residues was noted. A BLAST database search with the amino acid sequence of this annotated glycosyltransferase revealed it shares a high degree of sequence identity, 94.6 and 93.8% with UGT109A1 (Liang et al., 2017) and BsYjiC (Dai et al., 2017), respectively, two characterized UGTs from *B. subtilis* with catalytic specificity to diverse triterpenoids. This putative glycosyltransferase was designated as UGT109A3 after it was summited to the UGT Nomenclature Committee (Mackenzie et al., 1997).

**FIGURE 1:**
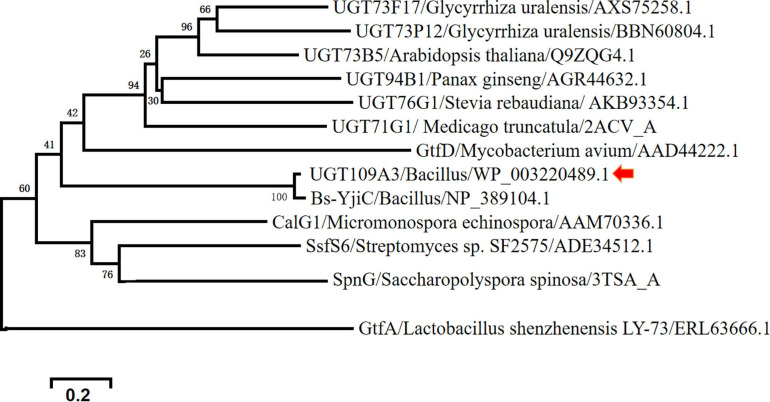
Phylogenetic relationships between UGT109A3 from *Bacillus subtilis* and other plant and microbial glycosyltransferases.

To verify the relationship of UGT109A3 to these other known UGT-family proteins, we constructed an evolutional relationship between various glycosyltransferase family homologs of plant and bacterial origin (Figure 1). This result confirms that the UGT109A3 protein from *Bacillus* is conserved as a homolog of the UGT family.

### Expression and Purification of Recombinant Enzymes

Determination of Enzymatic activity is essential to characterize the identified UGTs in sequenced genomes and to prove the predicted catalytic activity and specificity. The coding sequence of UGT109A3 was optimized, synthesized, and subcloned into a pET28a vector. The recombinant enzyme was expressed with N-terminal 6xHis tag in *E. coli* BL21 (*DE3*). To efficiently archive UGT109A3, we used an auto-induced expression system for the high-level production of recombinant enzymes. For auto-induced expression, *E. coli* rapid growth to high densities facilitates and ensures maximizing both folding efficiency and yield for producing target enzymes in the T7 expression system without added inducers (Studier, 2005). The recombinant UGT109A3 was expressed as a soluble protein and purified by Ni-NTA affinity chromatography. SDS-PAGE revealed a protein band corresponding to a molecular weight of around 43.0 kDa (Supplementary Figure 2A), that was consistent with the theoretically calculated molecular weight of 43.7 kDa for the 6xHis-tagged UGT109A3 protein. The UGT109A3 was found to exist as a monomer in solution, as revealed by gel-filtration chromatography (Supplementary Figure 3). The expression level of UGT109A3 was approximately 650 mg/L culture medium, which was much higher than those of plant UGTs involved in the GA glycosylation from previous studies (Liang et al., 2017).

### Glycosylation of Glycyrrhetinic Acid With UGT109A3

**FIGURE 2:**
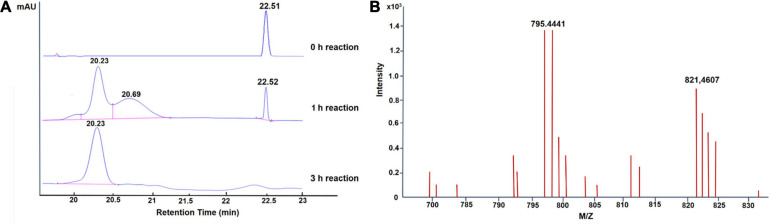
Enzymatic glycosylation of GA with UGT109A3. **(A)** HPLC analysis of glycosylation products; the elution was monitored at 254 nm. **(B)** LC–ESI-MS analysis of GA-glycoside products synthesized by UGT109A3. The mass data showed an (M + H)^+^ ion peak at m/z: 795.441 in the ESI-MS spectrum, corresponding to GA diglucoside product.

To assess the potential of GA glycosylation using UGT109A3, we investigated the UGT109A3 activity toward GA with UDPG acting as a sugar donor. After an incubation period of 3 h at 37°C, a new major peak with a retention time of 20.23 min was observed in HPLC (Figure 2A). This product was isolated from a preparative-scale reaction, and the HPLC–ESI–MS analysis revealed that the novel major product to be a diglucoside derivative of GA [Figure 2B, (M + H) + ion at m/z 795.444]. This glycosylated product of GA was further analyzed with 1H and 13C NMR (Supplementary Table 1). Compared with previously reported GA glycosylation (Dai et al., 2018a), the recombinant UGT109A3 enzyme was found to catalyze the transfer of the glucosyl moiety to both the free 3-OH and 30-COOH groups of GA. In the NMR spectra (Supplementary Figure 5), the significant downfield shift on the C3 (∼11.1 ppm) of GA backbone, along with the upfield shift of the anomeric carbon signal C1″ (∼11.0 ppm) suggested that glucosyl moieties were attached to both the C3 hydroxyl and the C30 carboxyl group of GA. The correlations of HMBC to the anomeric proton signal H1′ (δH 4.32, d, *J* = 7.80 Hz) with C3 (δC 90.3) and the anomeric proton signal H1″ (δH 5.52, d, *J* = 8.20 Hz) with C30 (δC 176.7) further revealed that the diglucoside derivative product was a unique 3, 30-O-β-D-diglucoside-GA.

**FIGURE 3:**
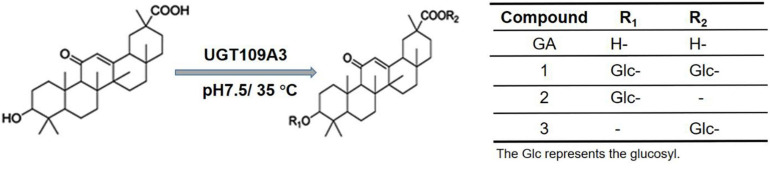
Chemical structure of GA and respective glycosylated products.

When the incubation period was shortened to 1 h, one additional small peak with retention time at 20.69 min was also observed (Figure 2A). The HPLC–ESI–MS analysis showed that it was a GA-monoglucoside, the intermediate product of GA glycosylation [Supplementary Figure 4; (M + H) + ion at m/z 655.372]. No obvious new retention peak was detected even if the reaction conditions were adjusted for the control reaction using lysate of *E. coli* containing an empty pET28a vector, confirming that these glycosylated products are from the UGT109A3-catalyzed reactions. The catalytic specificity of UGT109A3 is similar to several characterized UGTs, including UGT73C11 and BsYjiC, which could transform GA to 3-O-β-D-glucose-GA, 30-O-β-D-glucose-GA, and 3, 30-O-β-D-diglucoside-GA (Liu et al., 2017; Dai et al., 2018a). These results showed that UGT109A3 could serve as a promiscuous glycosyltransferase for the diverse glycosylation of acceptors (Figure 3).

To determine the affinity and catalytic efficiency, the kinetic parameters of UGT109A3 were determined by varying GA or UDPG substrate concentrations (Table 1). Compared with the kinetic parameters of UGT73C11 from *B. vulgaris* (Liu et al., 2017), the catalytic efficiencies of UGT109A3 against UDPG were considerably higher. This result further supported that UGT109A3 was an effective biocatalyst for glycosylation of nature products.

**TABLE 1:** Enzymatic kinetic parameters of UGT109A3.

Substrate	*K*m (mM)	*k*cat (s^–1^)
GA	0.52 ± 0.02	0.29 ± 0.14
UDPG	0.22 ± 0.01	59.67 ± 0.26

### Optimization of the UGT-SUS Cascade Biocatalysis for Producing GA Glycosides

**FIGURE 4:**
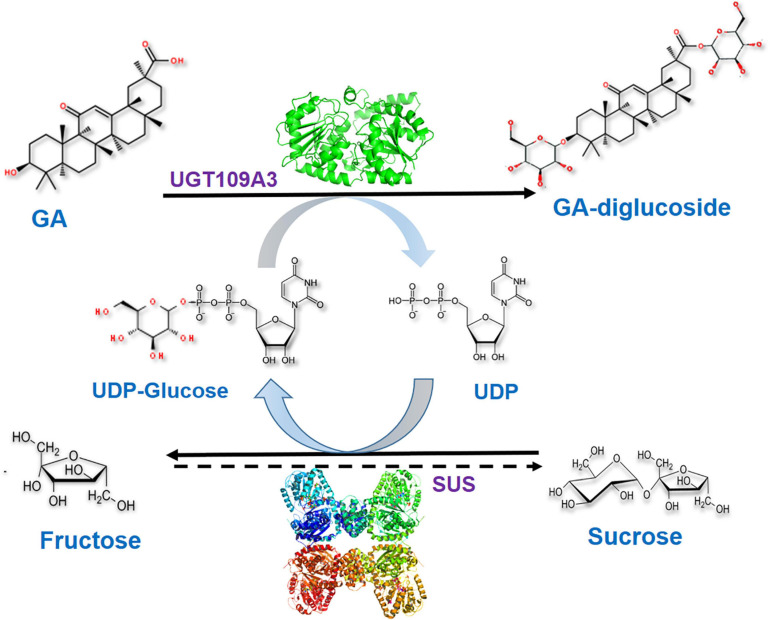
The biocatalytic cascade reaction catalyzed by UGT109A3 and SUS. The artificial cascade of UGT109A3-SUS potentially facilitates the conversion of GA into a 3, 30-O-β-D-diglucoside-GA, along with the costly UDPG sugar donor regeneration.

Glycosylation catalyzed by UGTs requires the costly UDPG as the donor thereby restricting the development of a scalable enzymatic glycosylation (Nidetzky et al., 2018). Therefore, the recombinant SUS from *A. thaliana* was also expressed in *E. coli* and purified to homogeneity (Supplementary Figure 2B). To produce GA-glucosides in an efficient and economical way, an artificial biocatalytic cascade of UGT109A3-SUS was constructed and optimized (Figure 4). The costly UDPG could be regenerated from UDP in the UGT109A3-SUS cascade reaction, along with the effective transformation of GA into GA glucosides. The enzyme ratio between UGT109A3 and SUS is one of the crucial factors for realizing synergistic cascade catalysis to obtain a high yield of GA-glucosides (Table 2). In the cascade reaction, with UGT109A3 concentration fixed at 30 μg/mL; only a 5.79% conversion rate increase was noted by changing the SUS concentration from 20 to 80 μg/mL. However, the optimal conversion rate could be raised approximately 200% by adding 50 μg/mL UGT109A3 and 20 μg/mL SUS in the reaction system, suggesting that UGT109A3 catalysis was the rate-limiting step in the cascade reaction. Thus, the optimal ratio between UGT109A3 and SUS could be set up at 5:2 for further experiments.

**TABLE 2:** Enzyme on the conversion rate of GA-glycosylated products*.

UGT1093 (μg/mL)	SUS (μg/mL)	Conversion rate (%)
30	20	56.93
30	40	58.45
30	60	60.19
30	80	62.72
10	20	45.57
20	20	48.49
30	20	55.24
40	20	79.85
50	20	93.48

*UGT109A3 and SUS were incubated with 0.5 mM GA, 0.4 mM UDP, and 500 mM sucrose in 100 mM Tris–HCl buffer pH 7.5 at 35°C for 1 h.

**FIGURE 5:**
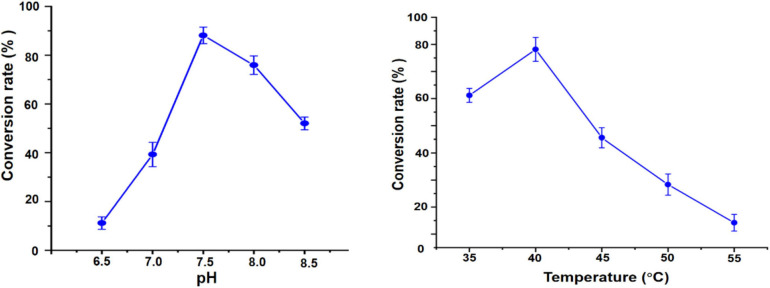
pH and temperature effects on the UGT109A3-SUS biocatalytic cascade reaction. Data represent the mean value ± standard deviation of three measurements.

**FIGURE 6:**
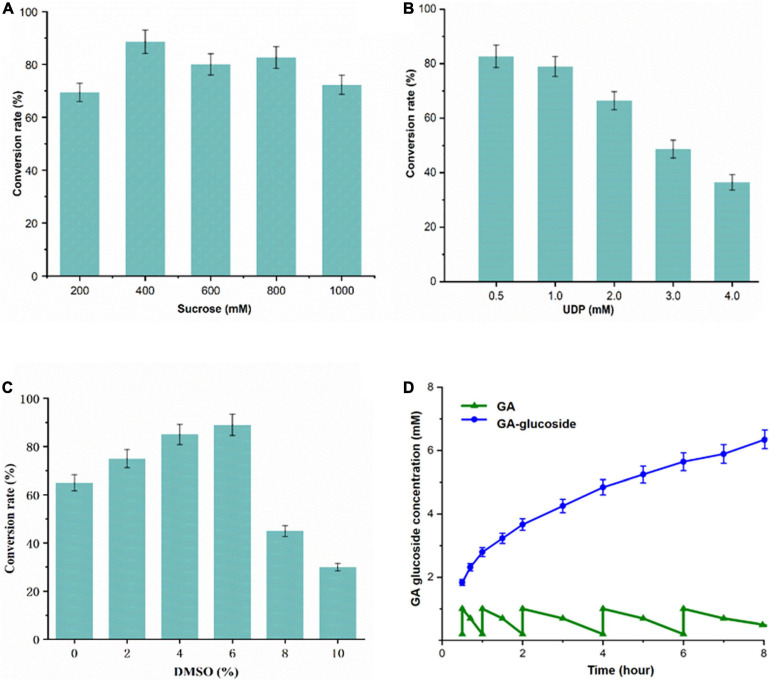
**(A–C)** Optimization of the UGT109A3-SUS biocatalytic cascade reactions and **(D)** fed-batch synthesis of GA glycosides. A total of 1 mM GA dissolved in DMSO was added stepwise to the reaction mixtures with 50 μg/mL UGT109A3 and 20 μg/mL SUS at 0.5, 1, 2, 4, 6, and 8 h. Fresh enzyme was added at 4 h. Data represents the mean value ± standard deviation of three measurements.

To optimize biocatalytic conditions, the effect of buffer pH and temperature on UGT109A3-SUS cascade activity was examined. Based on of GA-diglucoside yield, conditions of pH 7.5 and 40°C supported the most efficient catalysis (Figure 5). Considering that a lower temperature usually benefits enzyme stability, and 82% of the maximum activity could be reached at 35°C, the cascade reaction temperature was set at 35°C. The change in cascade catalysis efficiency with varying sucrose concentration was also investigated. The highest conversion rate was observed with the addition of 400 mM sucrose (Figure 6A). UDP is an essential substrate for SUS enzyme-mediated sucrose conversion to UDPG, but UDP is also a potent inhibitor of UGT glycosyltransferases (Terasaka et al., 2012). Thus, UDP complex effects in the UGT109A3-SUS cascade reactions were analyzed and a decline in conversion rates was observed with a minimum UDP concentration of 0.5 mM (Figure 6B).

Furthermore, the hydrophobic GA compound cannot dissolve well in an aqueous solution; therefore organic co-solvents could potentially facilitate the hydrophobic substrate glycosylation. The effects of organic solvents on the activity were examined. DMSO (<6% *v/v*) progressively promoted the glycosylation activity, whereas a higher concentration of DMSO strongly inhibited reaction (Figure 6C). Therefore, 35°C, pH 7.5, 0.4 mM UDP, 500 mM sucrose, and 1.0 mM GA were used for developing the UGT109A3-SUS cascade glycosylation.

### Fed-Batch Reaction for GA-Diglucoside Production

To realize a scale-up production of GA-glycosides, the UGT109A3-SUS cascade reaction was performed in 100 mL reaction volume. A fed-batch reaction was conducted by the periodic addition of 1.0 mM fresh GA. Under optimal conditions, the glycosylation occurred with almost near quantitative conversion (96%) of 5 mM GA acceptor substrate after a period of 5 h. After a longer 24 h reaction, GA could be converted entirely into 3, 30-O-β-D-diglucoside-GA as the sole product of the enzymatic glycosylation, facilitating the product isolations on a preparative scale. After 8 h, around 6.26 mM (4.98 g/L) GA-diglucoside was obtained via periodic feeding of GA into the reaction vessel (Figure 6D). These results proved that the UGT-SUS biocatalytic cascade has considerable potential for enzymatic glycosylation of GA.

### Solubility Improvement and Cytotoxicity of GA Glycosides

Many specific bioactivities of GA and GA derivatives have been reported. The poor aqueous solubility of natural products result in short retention time and poor bioavailability (Coimbra et al., 2011; Dewitte et al., 2016; Dymarska et al., 2018; Her et al., 2018). Glycosylation has been implemented to improve the solubility of hydrophobic natural products, as well as their pharmacodynamics (Nam et al., 2017). The effect of GA glycosylation on water solubility was evaluated (Table 3). Poor water solubility of GA was observed at 29 μM. The GA-diglucoside product, by contrast, was extremely effective in increasing the water solubility. A concentration of 1 × 10^5^ μM GA-diglucoside product was achieved in aqueous solution, an increase of around 3.4 × 10^3^ times compared to GA after the C3 and C30 carboxyl of GA were decorated with glucosyl moieties. This result was in agreement with previous studies that glycosylation could remarkably enhance the solubility of hydrophobic acceptors (De Bruyn et al., 2015; De Winter et al., 2015).

**TABLE 3:** Solubility of GA and GA-diglucoside in water.

Compounds	Water solubility		Fold
GA	13.6 μg/mL	29 μM	1
GA-diglucoside	8.1 × 10^4^ μg/mL	1 × 10^5^ μM	3.4 × 10^3^

**FIGURE 7:**
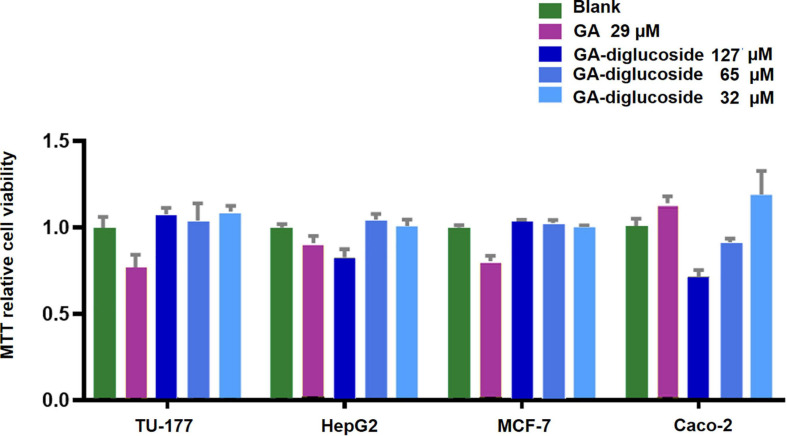
MTT Assay. Different cell lines were treated with GA or GA-diglucoside and then the cell viability was measured using the MTT assay. The result exhibited a dose-dependent cytotoxic of GA-diglucoside toward human colon carcinoma Caco-2 cell line, with an IC50 at 160 M.

Various nature products have been successfully glycodiversified, and some have shown enhanced biological properties compared to their respective parent molecules (Chen et al., 2012; Lepak et al., 2015). MTT assay revealed that the GA diglucoside derivatives presented *in vitro* dose dependent cytotoxic activity against human colon carcinoma Caco-2 cell line, with an IC_50_ value of 160 μM (Figure 7). However, this product was inactive toward human laryngeal cancer cell line Tu-177, liver cancer cell line HepG2, and breast cancer cell line MCF-7 (cut-off 200 μM). Yamaguchi et al. found that the cytotoxic effect of GA and GA derivatives was due to the downregulation of glutathione, disrupting the redox balance in those cells (Graebin, 2018). Xu et al. reported that GA derivatives induced the apoptosis in human myeloma U266 cell line by downregulating the surviving gene expression and arresting the cells in G0/G1 phase (Graebin, 2018). *In vivo* anticancer activity of GA-diglucoside should be studied further.

## Conclusion

In summary, the UGT109A3 from *B. subtilis* was identified as an efficient biocatalyst for glycosylation of GA, yielding 3, 30-O-β-D-diglucoside-GA. To efficiently biosynthesize GA glycosides, an artificial biocatalytic cascade with the glycosyltransferase UGT109A3 and SUS was constructed to realize the regeneration of costly sugar donor UDPG. The 3, 30-diglucoside GA, by contrast, is more water-soluble than GA. Cytotoxic activity f glycosylated GA against colon carcinoma Caco-2 cell line was also shown to be influenced by the type of sugar moiety attached to it. This study suggested that the *in vitro* UGT-SUS biocatalytic cascade reaction could be exploited as an efficient approach for large scale biosynthesis of triterpenoid saponins to improve their physical, chemical, and biological properties.

## Data Availability Statement

The datasets presented in this study can be found in online repositories. The names of the repository/repositories and accession number(s) can be found in the article/Supplementary Material.

## Author Contributions

QY and ZQ helped in the design and conducted the experiments. In addition, they helped in the interpretation of data for the work. KT and JW performed the data analysis. Besides, they helped in write the manuscript and Drafting the work or revising it critically. All authors read and approved the final manuscript.

## Conflict of Interest

The authors declare that the research was conducted in the absence of any commercial or financial relationships that could be construed as a potential conflict of interest.

## Footnotes

1 https://www.megasoftware.net/
