# Supplementary material for: Highly Efficient Biosynthesis of Glycyrrhetinic Acid Glucosides by Coupling of Microbial Glycosyltransferase to Plant Sucrose Synthase
Source: Front Bioeng Biotechnol. 2021 Jun 8;9:645079. doi: 10.3389/fbioe.2021.645079 (PMC8218813; doi:10.3389/fbioe.2021.645079)
Supplement: Supplementary file 1 [file Data_Sheet_1.pdf]

Supplementary Material

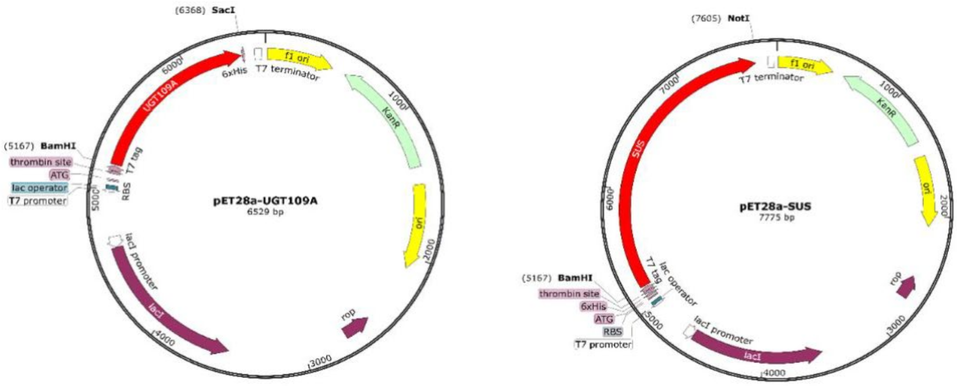

Figure S1. The constructed expression vectors. The expression vector of pET28a-UGT109A3 and pET28a-SUS were constructed for recombinant enzyme production.

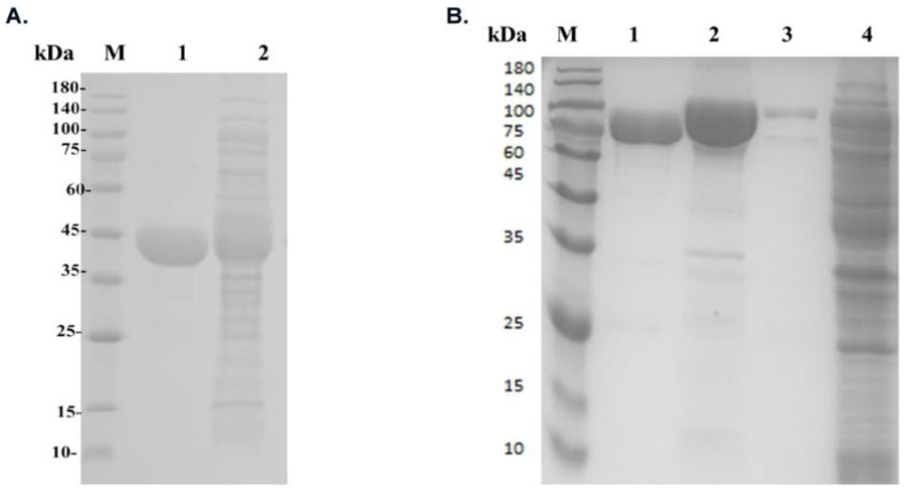

Figure S2. SDS-PAGE analysis of purified recombinant UGT109A3 and SUS enzymes. A. M: protein marker; lane 1: purified recombinant UGT109A3; lane 2: total lysates of *E. coli* BL21 (*DE3*) expressing UGT109A3. B. M: protein marker, Lane 1: purified recombinant SUS eluted with 120 mM imidazole buffer, lane 2: purified recombinant SUS eluted with 200 mM imidazole buffer; lane 3: processing with 30 mM imidazole washing buffer, lane 4: total lysates of *E. coli* BL21 (*DE3*) expressing recombinant SUS enzyme.

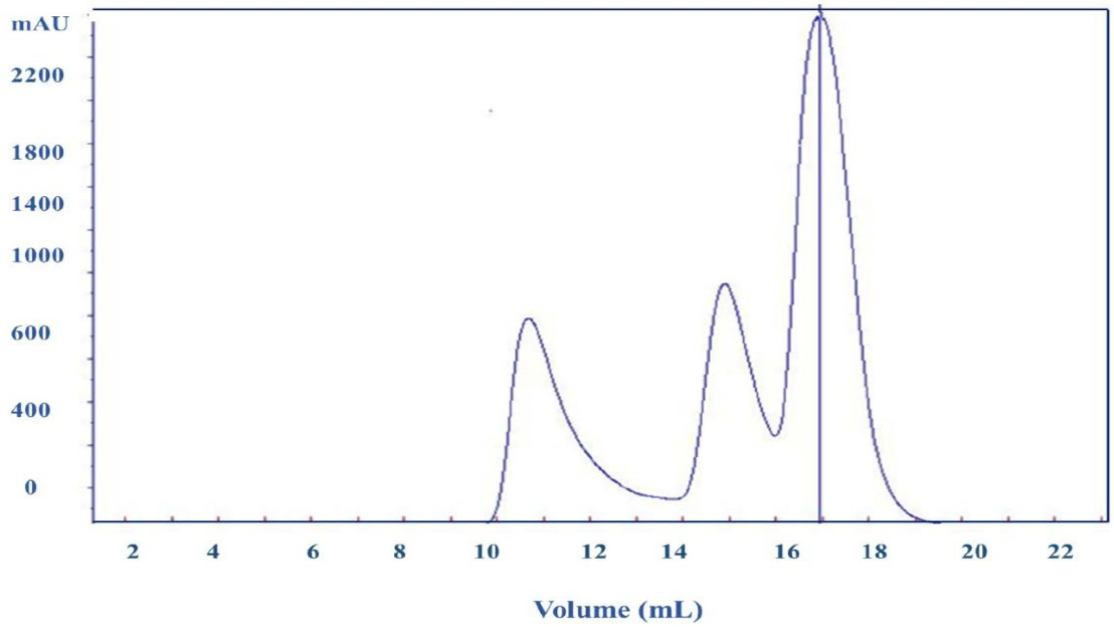

Figure S3. Gel filtration chromatography analysis of the 6xHis-tagged UGT109A3 recombinant protein.

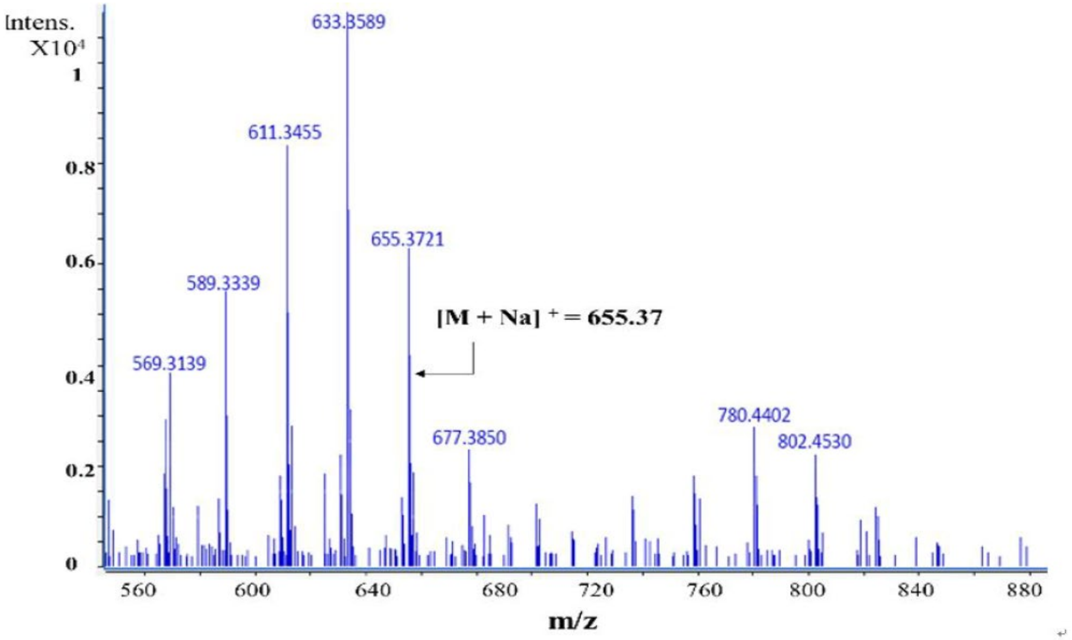

Figure S4. LC−ESI-MS analysis identified the monoglucoside GA products synthesized by UGT109A3. The mass data showed an $[M + Na]^+$ ion peak at m/z: 655.372 in the MS spectrum, corresponding to products GA-3-O-monoglucose or GA-30-O-monoglucose.

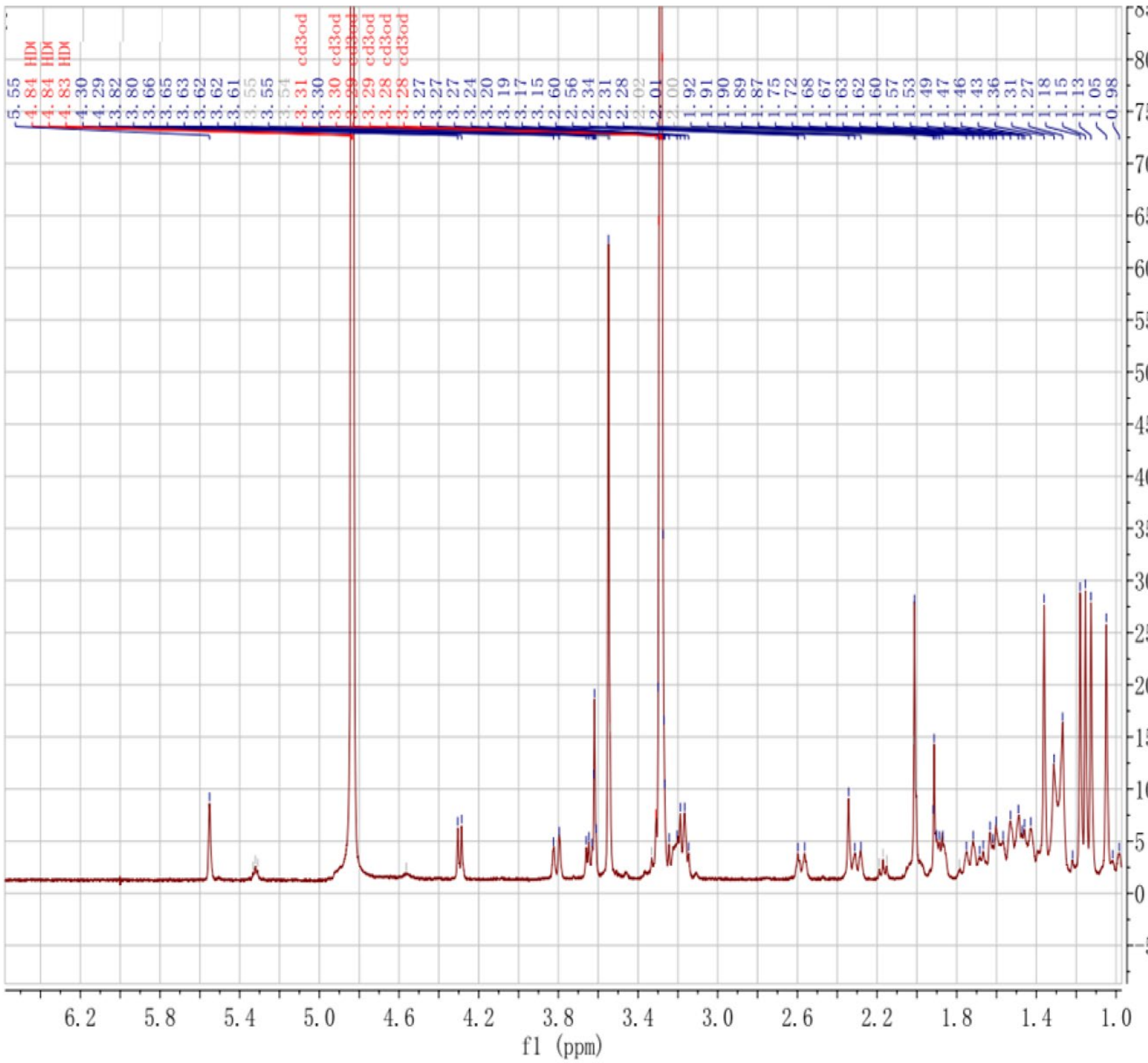

Figure S5. 1 H NMR spectra of the major product of GA glycosylation (Methanol-d4)

Table S1. 1H- and 13C-NMR spectral data for GA-diglucoside (Methanol-d4, 900 MHz)

C	δ_C	δ_H
1	40.3	CH_2, 1.02(m), 2.71(brd, *J*=13.60 Hz)
2	26.9	CH_2, 1.76(m), 1.91(m)
3	90.3	CH, 3.20(br,s)
4	40.3	
5	56.4	CH, 0.79(m)
6	18.4	CH_2, 1.48(m), 1.63(m)
7	33.7	CH_2, 1.46(m), 1.74(m)
8	46.5	
9	63.1	CH, 2.44(s)
10	38.1	
11	202.4	
12	128.9	CH, 5.62(s)
13	172.5	
14	44.3	
15	27.7	CH_2, 1.25(m), 1.89(m)
16	27.4	CH_2, 1.06(m), 2.15(td)
17	32.7	
18	49.3	CH, 2.25(m)
19	42.2	CH_2, 1.76(m), 1.91(m)
20	44.7	
21	31.9	CH_3, 1.47(m), 2.04(m)
22	38.5	CH_2, 1.40(m), 1.48(m)
23	28.5	CH_3, 1.08(s)
24	16.9	CH_3, 0.87(s)
25	17.0	CH_3, 1.15(s)
26	19.3	CH_3, 1.14(s)
27	23.8	CH_3, 1.42(s)
28	29.0	CH_3, 0.83(s)
29	28.2	CH_3, 1.21(s)
30	176.7	
3-O-Glc-1′	106.3	CH, 4.32(d, *J*=7.80 Hz)
3-O-Glc-2′	75.6	CH, 3.20(m)
3-O-Glc-3′	78.2	CH, 3.32(m)
3-O-Glc-4′	71.5	CH, 3.25(m)
3-O-Glc-5′	77.7	CH, 3.23(m)
3-O-Glc-6′	62.6	CH_2, 3.67(dd), 3.84(dd)
30-O-Glc-1″	95.6	CH, 5.52(d, *J*=8.20 Hz)
30-O-Glc-2″	74.0	CH, 3.34(m)
30-O-Glc-3″	78.3	CH, 3.43(m)
30-O-Glc-4″	71.1	CH, 3.39(m)
30-O-Glc-5″	78.9	CH, 3.40(m)
30-O-Glc-6″	62.5	CH_2, 3.71(dd), 3.86(dd)

Table S2. Primers used in plasmid construction.

Gene	Primer	Sequences (5' to 3')
**UGT109A3**	FP	CAGCGGTGGTGGTGGATCCATGAAAAAGCACCACATTA
(BamHI\XhoI)	RP	TGGTGGTGGTGGTGCTCGAGCTGCGGAACTGCGCTTT
**SUS**	FP	CGCGGATCCGCAAACGCTGAACGTATGATAACG
(BamHI\NcoI)	FP	CATGCCATGGTCAATCATCTTGTGCAAGAGGAACAGC

Restriction enzyme sites are underlined

Table S3. The amino acid sequence of UGT109A3 from Bacillus subtilis.

MKKHHISMINIPAYGHVNPTLALVEKLCEKGHRVTYATTEEFAPAVQQAGGEALIYH
TSLNIDPKQIREMMEKNDATLSLLKESLSILPQLEELYKDDQPDLIIYDFVALAGKLFA
DKLNVPVIKLCSSYAQNESFQLGNEDMLKKIKEAEAEFKAYLEQEQLPAVSFEQLAVP
EALNIVFMPKSFQIQHETFDDRFCFVGPSLGKRTEQESLLIDKGDRPLMLISLGTAFNA
WPEFYKMCIDAFRDSSWQVIMSVGKSIDPESLDDTPANFTIRQSVPQLEVLAKADLFIS
HGGMNSTMEAMNAGVPLVVIPQMYEQELTAKRVDELGLGVYLQREEVTVSKLQEA
VQAVSGDQELLSRVKSMQKDVKEAGGAERAAAEIEAFMKKSAVPQ

Table S4. The amino acid sequence of sucrose synthase from Arabidopsis thaliana.

MANAERMITRVHSQRERLNETLVSERNEVLALLSRVEAKGKGILQQNQIIAEFEALPQ
TRKKLEGGPFFDLLKSTQEAIVLPPWVALAVRPRPGVWEYLRVNLHALVVEELQPAE
FLHFKEELVDGVKNGNFTLELDFEPFNASIPRPTLHKYIGNGVDFLNRHLSAKLFHDK
ESLLPLLKFLRLHSHQGKNLMLSEKIQNLNTLQHTLRKAEEYLAELKSETLYEEFEAK
FEEIGLERGWGDNAERVLDMIRLLLDLLEAPDPCTLETFLGRVPMVFNVVILSPHGYF
AQDNVLGYPDTGGQVVYILDQVRALEIEMLQRIKQQGLNIKPRILILTRLLPDAVGTT
CGERLERVYDSEYCDILRVPFRTEKGIVRKWISRFEVWPYLETYTEDAAVELSKELNG
KPDLIIGNYSDGNLVASLLAHKLGVTQCTIAHALEKTKYPDSDIYWKKLDDKYHFSC
QFTADIFAMNHTDFIITSTFQEIAGSKETVGQYESHTAFTLPGLYRVVHGIDVFDPKFNI
VSPGADMSIYFPYTEEKRRLTKFHSEIEELLYSDVENKEHLCVLKDKKKPILFTMARL
DRVKNLSGLVEWYGKNTRLRELANLVVVGGDRRKESKDNEEKAEMKKMYDLIEEY
KLNGQFRWISSQMDRVRNGELYRYICDTKGAFVQPALYEAFGLTVVEAMTCGLPTFA
TCKGGPAEIIVHGKSGFHIDPYHGDQAADTLADFFTKCKEDPSHWDEISKGGLQRIEE
KYTWQIYSQRLLTLTGVYGFWKHVSNLDRLEARRYLEMFYALKYRPLAQAVPLAQD
D
